# Transabdominal Ultrasound Measured Rectal Diameter for Identifying Fecal Impaction in Children With Functional Constipation: A Diagnostic Accuracy Study

**DOI:** 10.7759/cureus.87100

**Published:** 2025-07-01

**Authors:** Keerthi Sunkari, Mohd Saeed Siddiqui, P S Mishrikotkar, Madhuri B Engade, R J Totla, Pradnya M Joshi, Sesha Sarat Chandra, Anju M Varghese

**Affiliations:** 1 Pediatrics, Mahatma Gandhi Mission (MGM) Medical College and Hospital, MGM Institute of Health Sciences, Aurangabad, IND; 2 Radiology, Mahatma Gandhi Mission (MGM) Medical College and Hospital, MGM Institute of Health Sciences, Aurangabad, IND; 3 Pediatric Surgery, Mahatma Gandhi Mission (MGM) Medical College and Hospital, MGM Institute of Health Sciences, Aurangabad, IND

**Keywords:** fecal impaction, functional constipation, rectal diameter, rectal wall thickness, transabdominal ultrasound

## Abstract

Background

Functional constipation (FC) is a common pediatric disorder, with fecal impaction posing significant diagnostic challenges. Digital rectal examination (DRE), to identify fecal mass in the rectum, is invasive and often distressing. Transabdominal ultrasound (TAUS) offers a promising, non-invasive alternative by measuring transverse rectal diameter (RD). This study was conducted to evaluate the diagnostic accuracy of ultrasound-measured RD in detecting fecal impaction among children with FC, using DRE as the reference standard.

Methods

In this diagnostic accuracy study conducted over 18 months, 80 children aged six months to 18 years with FC were recruited and divided into impaction (n=40) and non-impaction (n=40) groups. All cases underwent clinical evaluation, DRE, and blinded TAUS assessment. Mean RD was compared between the two groups, and subgroup analysis was performed to compare mean RD across age groups and fecal impaction status. Receiver operating characteristic (ROC) analysis was used to determine optimal RD cutoffs for diagnosing fecal impaction across two age groups (<4 years and >4 years).

Results

The mean transverse rectal diameter was significantly larger in the impaction group (mean = 3.38 cm, SD = 1.32) compared to the non-impaction group (mean = 2.15 cm, SD = 0.93), indicating a large effect size (Cohen’s d = 1.07, p < 0.001). The subgroup >4 years with fecal impaction had significantly larger RD (mean = 3.62, SD = 1.49) compared to the same age group without fecal impaction (mean = 2.1, SD = 1.16, p < 0.001). ROC analysis showed moderate accuracy for an RD cut-off of 2.5 cm in children <4 years (sensitivity 60%, specificity 68.75%, AUC = 0.616) and higher accuracy at a 3.5 cm cut-off for children >4 years (sensitivity 63.3%, specificity 95.8%, AUC = 0.803). Rectal wall thickness did not significantly differ between the groups.

Conclusion

Ultrasound-measured RD is a valuable, non-invasive diagnostic tool for identifying fecal impaction in pediatric functional constipation, particularly in children over four years, when using age-specific cut-off values. Larger multicentric studies are required to confirm and refine the cut-off values. Incorporating transabdominal ultrasound into routine practice may enhance patient comfort while maintaining diagnostic accuracy.

## Introduction

Functional constipation (FC) is a common pediatric gastrointestinal disorder, affecting approximately 9.5% of children worldwide, and is characterized by infrequent, difficult, or painful defecation without an identifiable organic cause [1]. One of the most clinically significant associated problems with FC is fecal impaction, defined as the accumulation of large, hardened stools in the rectum, which can exacerbate symptoms such as abdominal pain, fecal incontinence, and urinary disturbances, profoundly affecting the child’s quality of life [2].

The diagnosis of FC is primarily based on the Rome IV criteria, which emphasize clinical symptoms alongside, where necessary, confirmation of fecal retention via digital rectal examination (DRE) [3]. However, DRE is invasive, often distressing for children, and subject to examiner variability, limiting its acceptability and reliability in routine pediatric practice [4]. Similarly, abdominal radiography and colonic transit studies, although available, carry disadvantages such as radiation exposure, poor reproducibility, and limited child-friendliness [5,6].

Transabdominal ultrasonography (TAUS) has emerged as a promising non-invasive diagnostic alternative, enabling assessment of rectal diameter (RD) and fecal loading. Studies consistently report that children with FC exhibit significantly larger rectal diameters compared to healthy peers [7,8]. A recent systematic review and meta-analysis estimated pooled sensitivity and specificity for TAUS in diagnosing constipation at 68% and 81%, respectively [9]. Furthermore, TAUS offers the advantage of serial monitoring of treatment response by documenting reductions in rectal diameter following laxative therapy [10].

Despite these advantages, several limitations restrict the widespread clinical adoption of TAUS. Existing studies demonstrate substantial heterogeneity in study designs, patient populations, and RD cut-off values for diagnosing fecal impaction, which have ranged from 2.4 to 3.8 cm [11]. Moreover, factors such as the time since last defecation and prior laxative use significantly influence RD measurements, necessitating standardized protocols [12].

This study aims to evaluate the diagnostic utility of ultrasound-measured RD in detecting fecal impaction among children with FC. Specifically, it seeks to assess diagnostic accuracy in two age groups. This study may help to strengthen the evidence base for incorporating TAUS as a reliable, non-invasive diagnostic modality in the routine management of pediatric constipation.

## Materials and methods

Study design and setting

This diagnostic accuracy study was conducted over 18 months (May 2023 to October 2024) at the outpatient department of a tertiary care hospital in Maharashtra, India. The study was designed to assess the diagnostic performance of transabdominal ultrasound-measured transverse rectal diameter for detecting fecal impaction in children with functional constipation, using digital rectal examination as a reference standard.

Study population

Children aged 6 months to 18 years diagnosed with FC based on the Rome IV criteria [3] were eligible for inclusion. Diagnosis required the presence of at least two symptoms, such as ≤ two bowel movements per week, fecal incontinence, retentive posturing, passage of large-diameter stools, or hard/painful stools, persisting for a minimum duration of one month. Exclusion criteria were known organic causes of constipation (e.g., Hirschsprung disease, hypothyroidism, spinal anomalies), prior rectal or abdominal surgery, neurological disorders affecting bowel function, recent acute gastrointestinal illness (within the past four weeks), or refusal to undergo digital rectal examination or ultrasound. For subgroup analysis, age groups of <4 years and >4 years were chosen, as the Rome IV criteria use this cut point in the diagnosis of FC.

For the comparison of RD, G*Power software was used, assuming a standardized effect size of 0.8 (based on a ~1.0 cm RD difference and pooled SD of 1.25 cm from previous studies), α = 0.05, and 90% power, yielding 34 children per group (total 68) [7,9]. Accounting for a 10% dropout rate, the adjusted sample size for the RD comparison was a total of 76. For AUC-ROC analysis, the Cleveland Clinic sample size calculator [13] was employed, assuming an AUC of 0.85 [9], α = 0.05, 95% power, and a 1:1 allocation ratio, requiring 20 children per group (total 40). For two age groups, a sample size of 80 (40 each for with and without impaction groups based on the presence or absence of a hard, large fecal mass on DRE) was chosen to meet power for both the comparison of RD and ROC analysis.

Ethical considerations

Participants were recruited consecutively during routine outpatient visits. Informed written consent was obtained from parents or legal guardians. Assent was obtained from children aged ≥7 years. The study has been approved by the Institutional Ethics Committee (Approval No.: MGM-ECRHS/2023/44, Dated: 27/04/2023).

Data collection

All participants underwent clinical assessment by a pediatrician to confirm the diagnosis according to the Rome IV criteria. Clinical data included demographics, symptom duration, bowel movement frequency, stool consistency (Bristol Stool Form Scale), laxative use, and time since last defecation. DRE was conducted by a trained pediatrician blinded to ultrasound findings. Fecal impaction was defined as the presence of hard, immobile stool palpable on digital rectal examination [2]. All the children with functional constipation underwent DRE. The procedure was explained to the child and caregiver in an age-appropriate manner, informed consent was obtained, and the examination was conducted in a private setting with the child positioned in the lateral decubitus position. A trained pediatrician performed the DRE using a lubricated, gloved finger (typically the smallest digit), gently inserted into the rectum to evaluate fecal mass, while ensuring the child’s comfort through continuous monitoring and, if necessary, distraction techniques. The children were divided into two groups of 40 each, those with fecal impaction and those without, based on the presence or absence of a large, hard fecal mass detected during digital rectal examination (DRE).

Ultrasound was performed by certified radiologists using a high-resolution Voluson E8 machine (GE HealthCare, India), equipped with linear (2-14 MHz) and curvilinear (1-7.5 MHz) transducers. Children were asked to empty their bladders one hour prior to the scan. Scanning was performed in supine and left lateral decubitus positions to optimize visualization. Rectal diameter was measured in the transverse plane at the rectal ampulla, defined as the widest anteroposterior diameter perpendicular to the lumen. Also, the anterior rectal wall thickness was measured at the same location. The radiologist was blinded to clinical and DRE data.

Statistical analysis

Continuous variables were reported as mean ± SD, and categorical variables as frequency and percentage. Transverse rectal diameters and anterior rectal wall thickness were compared between the two groups using an independent t-test. Analysis of covariance (ANCOVA) test was used to compare mean RD across four subgroups of age group by fecal impaction status. Diagnostic accuracy was expressed as sensitivity, specificity, positive predictive value (PPV), and negative predictive value (NPV), with DRE as the reference standard, using ROC analysis, and a curve was plotted to determine optimal RD cut-off values for the two age groups. Analyses were conducted using Jamovi software version 2.4.1, and p < 0.05 was considered statistically significant [14].

## Results

This study investigated the utility of ultrasound-measured transverse rectal diameter and rectal wall thickness in diagnosing fecal impaction among children with functional constipation, stratified into groups with and without impaction. By assessing demographic characteristics, clinical features, and diagnostic accuracy through statistical analyses including t-tests and ROC curves, this study aimed to evaluate the potential of ultrasound as a reliable, patient-friendly diagnostic tool, with a focus on age-specific thresholds.

The demographic analysis of 80 children with functional constipation, comprising 40 with and 40 without fecal impaction, revealed no significant differences in age (mean 7.3 ± 4.5 years, p = 0.081) or sex distribution, with 36 (45.0%) females and 44 (55.0%) males (p = 1.000), indicating a balanced cohort across groups. Weight (mean 21.0 ± 11.3 kg, p = 0.225) and height (mean 113.3 ± 26.3 cm, p = 0.081) also showed no significant variation. Notably, clinical features such as two or fewer defecations per week, 22 (55.0%) vs. 16 (40.0%) (p = 0.179); history of excessive stool retention, 26 (65.0%) vs. 21 (52.5%) (p = 0.256); and painful hard movements, 33 (82.5%) vs. 35 (87.5%) (p = 0.531), were prevalent but not significantly different between groups. A significantly higher number of children, 26 (76.5%), in the fecal impaction group had at least one episode of fecal incontinence in a week as compared to the no-impaction group, 8 (23.5%) (p < 0.001). Also, the presence of palpable fecoliths 35 (87.5%) vs. 14 (35.0%) was significantly more common (p < 0.001) in the fecal impaction group (Table 1).

**Table 1:** Demographic and clinical characteristics of fecal impaction vs. no impaction groups

Variable	No Impaction (N=40)	Impaction (N=40)	Total (N=80)	p-value
Age (Years)				0.081
Mean (SD)	6.4 (4.6)	8.2 (4.3)	7.3 (4.5)	
Range	0.8 - 16.6	1.6 - 17.5	0.8 - 17.5	
Sex n (%)				1
Female	18.0 (45.0)	18.0 (45.0)	36.0 (45.0)	
Male	22.0 (55.0)	22.0 (55.0)	44.0 (55.0)	
Weight(kg)				0.225
Mean (SD)	19.4 (11.4)	22.5 (11.1)	21.0 (11.3)	
Range	7.0 - 49.0	7.0 - 49.0	7.0 - 49.0	
Height (cm)				0.068
Mean (SD)	108.0 (27.4)	118.7 (24.2)	113.3 (26.3)	
Range	60.0 - 162.0	80.0 - 163.0	60.0 - 163.0	
Two or Few Defecations Per Week n(%)				0.179
No	22.0 (55.0)	16.0 (40.0)	38.0 (47.5)	
Yes	18.0 (45.0)	24.0 (60.0)	42.0 (52.5)	
Excessive Stool Retention n (%)				0.256
No	14.0 (35.0)	19.0 (47.5)	33.0 (41.2)	
Yes	26.0 (65.0)	21.0 (52.5)	47.0 (58.8)	
At least One Incontinence Episode/wk n (%)				<0.001
No	16 (80)	4 (20)	20 (37)	
Yes	8 (23.5)	26 (76.5)	34 (63)	
Painful Hard Movements n (%)				0.531
No	7.0 (17.5)	5.0 (12.5)	12.0 (15.0)	
Yes	33.0 (82.5)	35.0 (87.5)	68.0 (85.0)	
Large Diameter Stools n (%)				0.823
No	20.0 (50.0)	21.0 (52.5)	41.0 (51.2)	
Yes	20.0 (50.0)	19.0 (47.5)	39.0 (48.8)	
Fecoliths Palpable n (%)				< 0.001
No	26.0 (65.0)	5.0 (12.5)	31.0 (38.8%)	
Yes	14.0 (35.0)	35.0 (87.5)	49.0 (61.2%)	

**Figure 1:**
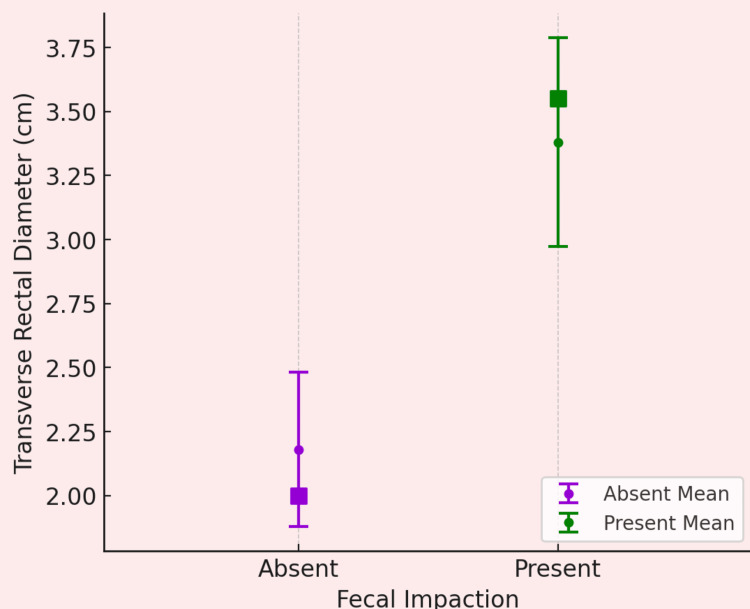
Comparison of mean rectal diameter between fecal impaction and no impaction groups

The independent samples t-test analysis demonstrated significant differences in transverse rectal diameter but not in rectal wall thickness (Table 2). The mean transverse RD was significantly larger in the impaction group (mean = 3.38 cm, SD = 1.32) compared to the non-impaction group (mean = 2.15 cm, SD = 0.93) (Figure 1), indicating a large effect size (Cohen’s d = 1.07, p < 0.001). The Welch’s t-test was used due to unequal variances (Levene’s test, p < 0.05). In contrast, rectal wall thickness showed no significant difference between the two groups, with a mean difference of 0.307 mm (Student’s t = 1.45, df = 78, p = 0.152), suggesting limited diagnostic utility for this parameter.

**Table 2:** Comparison of transverse rectal diameter and rectal wall thickness between fecal impaction and no impaction groups df: Degree of freedom, SE: Standard error, CI: Confidence interval, Cohen's d: standardized mean difference

Variable	Impaction Group Mean (SD)	No Impaction Group Mean (SD)	t statistic	df	p value	Mean Difference	SE of Difference	95% CI	Cohen's d
Transverse Rectal Diameter (cm)	3.38 (1.32)	2.15 (0.93)	4.8	70.4	<0.001	1.225	0.255	[0.716, 1.734]	1.074
Rectal Wall Thickness (mm)	2.94 (1.05)	2.63 (0.84)	1.45	78	0.152	0.307	0.213	[-0.116, 0.731]	0.323

**Figure 2:**
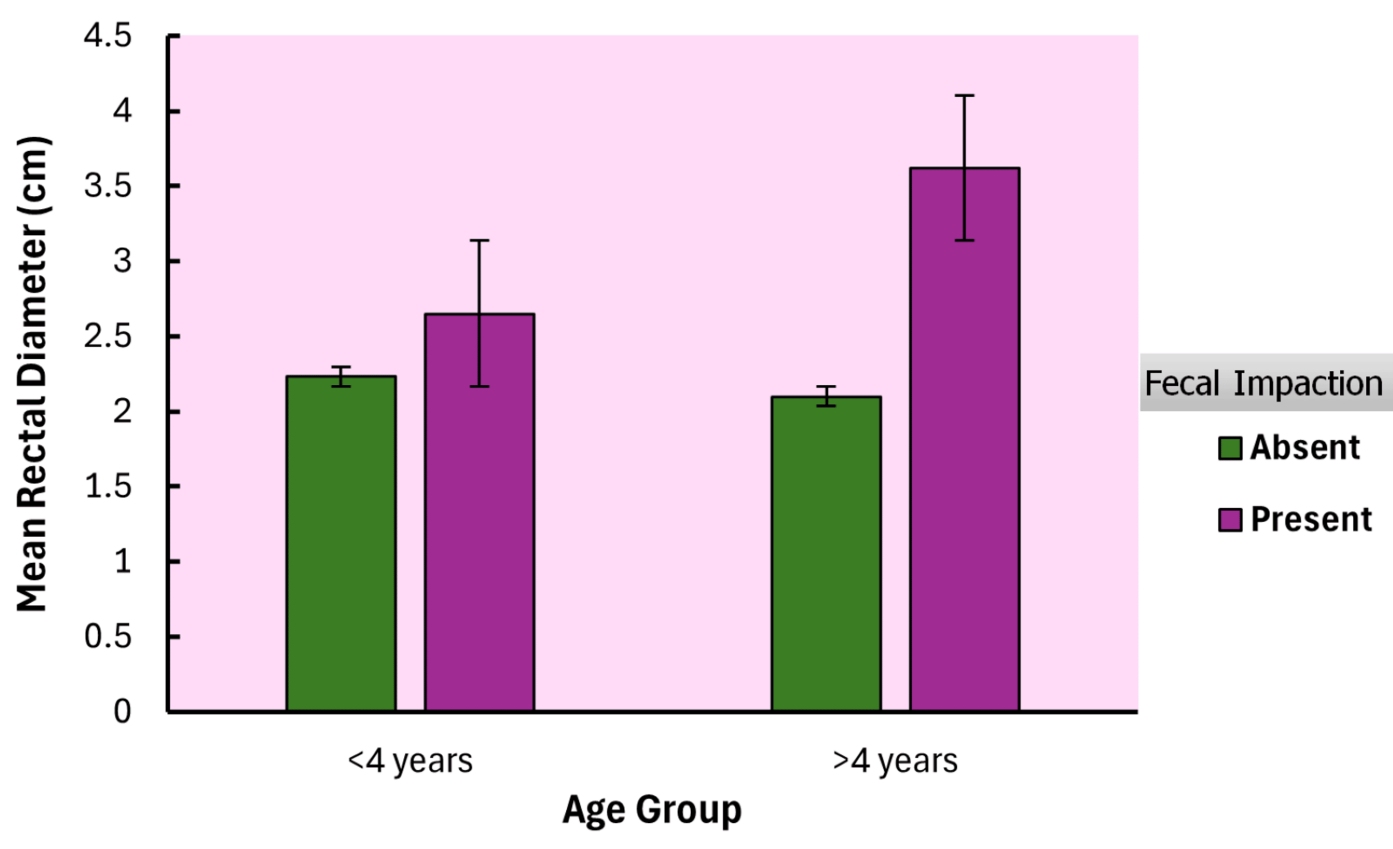
Comparison of mean rectal diameter across subgroups by age and fecal impaction status

Analysis of covariance (ANCOVA) test was used to compare mean RD across four subgroups of age group by fecal impaction status (Table 3). In the >4 years group, rectal diameter was significantly larger with fecal impaction (mean = 3.62, SD = 1.49) compared to no impaction (mean = 2.1, SD = 1.16), with a strong effect size (p < 0.001, Cohen's d = -1.365). No significant differences were observed within the four-year no-impaction groups (p = 0.983, Cohen's d = 0.118). A trend toward significance was noted between the >4 years with impaction subgroup (mean = 3.62, SD = 1.49) and the <4 years with impaction subgroup, with a large effect size (p = 0.089, Cohen's d = -0.871). Figure 2 shows the comparison of the subgroups.

**Table 3:** Comparison of mean rectal diameter by age group and fecal impaction status Subgroups were <4:0 = <4 years without fecal impaction, <4:1 = <4 years with fecal impaction, >4:0 = >4 years without fecal impaction, and >4:1 = >4 years with fecal impaction; SE: Standard error

Subgroup	Mean (SD)	Subgroup	Mean (SD)	Mean Difference	SE of difference	df	t statistic	p-value	Cohen's d
<4:0	2.23 (1.42)	<4:1	2.65 (2.59)	-0.419	0.449	76	-0.933	0.787	-0.376
<4:0	2.23 (1.42)	>4:0	2.1 (1.16)	0.131	0.359	76	0.365	0.983	0.118
<4:1	2.65 (2.59)	>4:1	3.62 (1.49)	-0.97	0.407	76	-2.385	0.089	-0.871
>4:0	2.1 (1.16)	>4:1	3.62 (1.49)	-1.52	0.305	76	-4.983		-1.365

**Figure 3:**
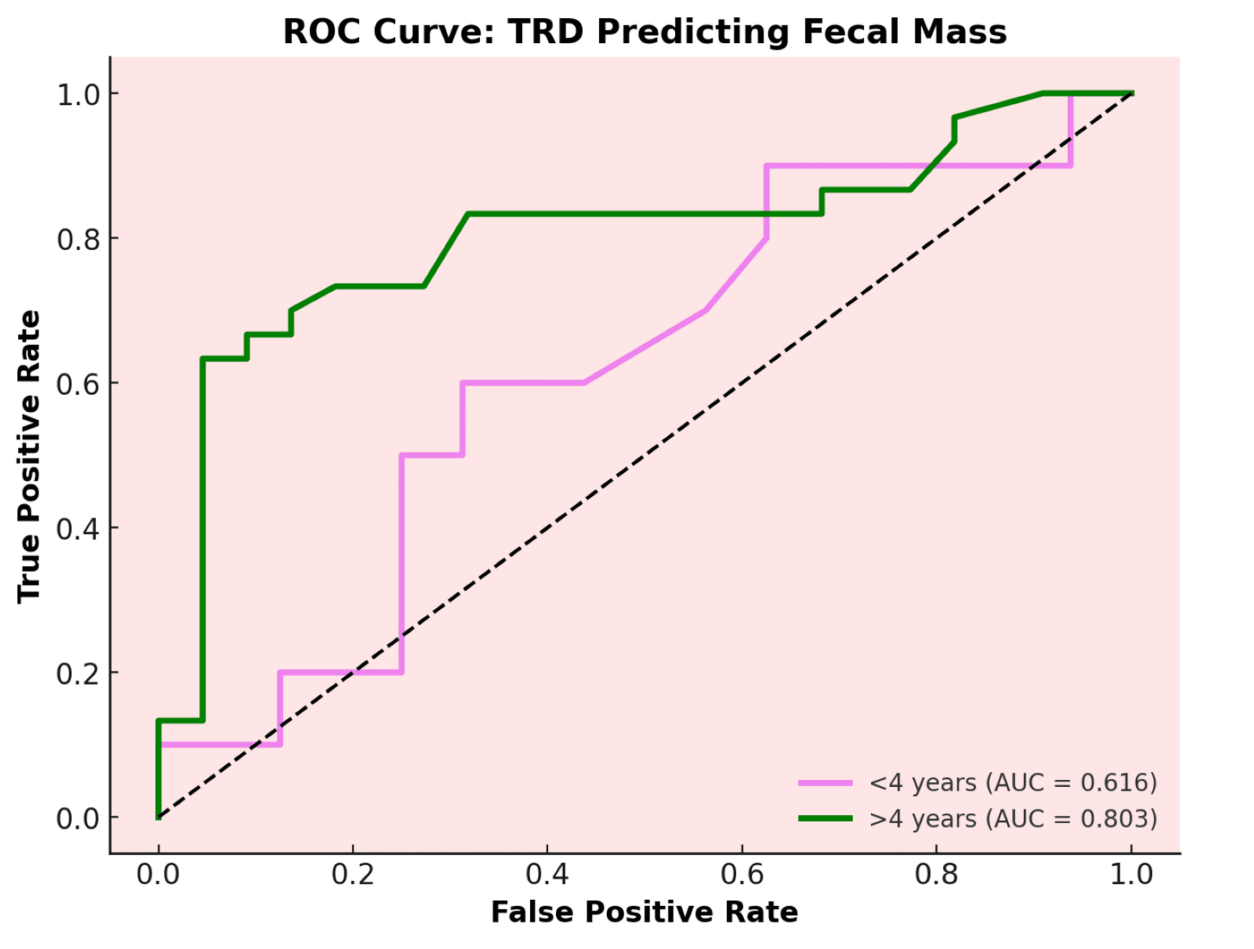
Receiver operating curve of rectal diameter to diagnose fecal impaction ROC: Receiver operating curve, TRD: Transverse rectal diameter

The ROC analysis for transverse RD in diagnosing fecal impaction across 80 children, stratified by age groups (<4 years and >4 years), revealed distinct diagnostic performance thresholds (Table 4). For children under 4 years, a cutpoint of 2.5 cm demonstrated a sensitivity of 60% and specificity of 68.75% (AUC = 0.616), indicating moderate discriminatory ability. In contrast, for children over 4 years, a cutpoint of 3.5 cm yielded a higher sensitivity of 63.3% and specificity of 95.8% (AUC = 0.803), suggesting higher discriminatory ability in this age group. These findings highlight the potential benefit of age-specific cutpoints, with 2.5 cm serving as an effective threshold for younger children and 3.5 cm offering enhanced specificity and predictive value for older children (Figure 3).

**Table 4:** Diagnostic performance of rectal diameter cutpoints for fecal impaction by age group PPV: Positive predictive value, NPV: Negative predictive value, AUC: Area under the ROC curve, Metric Score: Composite score reflecting overall diagnostic utility

Age Group	Cutpoint (cm)	Sensitivity (%)	Specificity (%)	PPV (%)	NPV (%)	Youden's index	AUC	Metric Score
<4 years	2.5	60	68.75	54.55	73.33	0.288	0.616	1.29
>4 years	3.5	63.33	95.83	95	67.65	0.592	0.803	1.59

## Discussion

Functional constipation is a common pediatric condition, often complicated by fecal impaction, which poses diagnostic challenges due to the invasiveness of traditional methods like digital rectal examination. Transabdominal ultrasound, measuring transverse RD and rectal wall thickness, offers a non-invasive alternative to detect rectal enlargement associated with FC and fecal impaction. This study aimed to identify the diagnostic utility of ultrasound-measured RD and rectal wall thickness in children with FC, stratified into groups with and without fecal impaction.

Transverse RD measurement has demonstrated utility in diagnosing FC in children, as evidenced by a systematic review and meta-analysis of 16 studies (n = 1,801 children) [9]. The meta-analysis reported a pooled sensitivity of 68% (95% CI 0.55-0.78) and specificity of 81% (95% CI 0.71-0.88) for RD in differentiating children with FC from controls (standardized mean difference = 1.37, p < 0.0001), with cut-off values ranging from 2.4 cm to 3.8 cm. Key studies highlighted that children with FC exhibited significantly larger RD correlating with chronic fecal retention [7,15]. While an RD threshold of 3.0 cm was commonly used, variability in measurement protocols (e.g., bladder filling state, transducer placement) and lack of age-stratified norms limited clinical standardization [9]. For instance, Pop reported an optimal RD cut-off of 3.0 cm (sensitivity: 68%, specificity: 81%), whereas Hamdy proposed a lower threshold of 2.8 cm for younger children [11,16]. These findings underscore RD’s potential as a non-invasive diagnostic tool, though age-specific protocols are needed to improve reproducibility [9]. 

We found that the mean transverse RD was significantly larger in the impaction group compared to the non-impaction group, with a mean difference of 1.225 cm (95% CI: 0.716 cm to 1.734). Vos et al., in their meta-analysis, evaluated RD’s accuracy for fecal impaction detection, though heterogeneity precluded pooled analysis [9]. Individual studies reported sensitivity and specificity ranges of 68-100% and 83-100%, respectively, with RD cut-offs between 2.7 cm and 3.0 cm [8,17]. Joensson identified an RD threshold of 2.94 cm with 100% sensitivity and specificity for impaction, while Burgers reported a 3.0 cm cut-off (sensitivity 68%, specificity 92%) [15,18]. Longitudinal data from Modin-Walsted et al. demonstrated that RD decreased significantly in post-disimpaction (mean reduction: 1.2 cm), supporting its role in monitoring treatment efficacy [17]. However, inconsistencies in defining fecal impaction (e.g., reliance on DRE vs. clinical criteria) and variability in measurement techniques (e.g., bladder filling protocols) reduced comparability across studies [8,19]. These limitations highlight the need for standardized definitions and age-adjusted RD thresholds to enhance diagnostic reliability [9]. 

In our study, rectal wall thickness showed no significant difference, with a mean difference of 0.307 mm (95% CI: -0.116 to 0.731). Recent studies highlight the potential of transabdominal ultrasound in assessing rectal wall thickness for diagnosing functional constipation and fecal impaction in children, though findings vary by measurement focus. Doğan found significantly thicker anterior rectal walls in constipated children, especially with fecal mass (e.g., 2.24 ± 0.84 mm in 6.1-12 years, p < 0.0001), correlating with constipation duration (r = 0.40, p = .000) [20]. Conversely, Momeni observed thinner overall rectal walls in constipated children (1.75 ± 0.33 mm vs. 1.90 ± 0.22 mm in controls, p = 0.032) [21], and in another study, the thickness of the anterior wall did not correlate with the presence of fecal incontinence (r = 0.02; p = 0.39), suggesting measurement discrepancies [11]. These results underscore ultrasound’s non-invasive diagnostic value but emphasize the need for standardized protocols to clarify anterior versus overall wall thickness changes and enhance clinical reliability.

In the subgroup analysis, the >4 years group, rectal diameter was significantly larger with fecal impaction compared to no impaction. No significant differences were observed within the four-year no-impaction groups. A trend toward significance was noted between the >4 years with impaction subgroup and the <4 years with impaction subgroup, with a large effect size. The ROC analysis in our study for transverse RD in diagnosing fecal impaction, stratified by age, revealed distinct performance thresholds. For children under 4 years, a cut point of 2.5 cm showed a sensitivity of 60% and specificity of 68.75% (AUC = 0.616), indicating moderate discriminatory ability. For children over 4 years, a cut point of 3.5 cm demonstrated a higher sensitivity of 63.3% and specificity of 95.8% (AUC = 0.803), suggesting superior diagnostic accuracy. Dogan reported age-dependent increases in RD, with mean values rising from 2.8 cm (±0.5) in children <3 years to 3.9 cm (±1.1) in those >12 years, reinforcing the correlation between age and rectal diameter [20]. Similarly, Hamdy and Pop proposed age-adjusted RD thresholds (e.g., 2.8 cm for younger children vs. 3.1 cm for older cohorts) but did not formally stratify their cohorts [11,16]. In contrast, most studies analyzed RD as a continuous variable across broad age ranges (0-17 years), with others noting a positive age-RD correlation [11,22]. For instance, Singh observed a larger RD in older children (median 3.4 cm vs. 2.4 cm in controls) but lacked age-specific cutoffs [10]. Overall, while age-related RD trends were acknowledged, only two studies systematically stratified analyses, highlighting the meta-analysis’s call for standardized, age-specific protocols to improve diagnostic precision [9]. 

This study has several limitations. The relatively small sample size may limit the generalizability of the findings, particularly for age-stratified analyses. Variability in ultrasound measurement techniques, such as differences in bladder filling state or transducer placement, could introduce inconsistencies in transverse RD and rectal wall thickness assessments. The reliance on DRE for defining fecal impaction may introduce subjectivity, potentially affecting diagnostic accuracy. Additionally, the lack of longitudinal data precludes evaluation of ultrasound’s utility in monitoring treatment outcomes. Finally, the study’s focus on a single center may not account for diverse patient populations or clinical practices, warranting caution in applying results to broader settings.

Future multicentric research should prioritize developing standardized ultrasound protocols, including consistent bladder filling and measurement techniques, and establish age-specific RD and RWT cutoffs through large, prospective studies with well-defined age cohorts. Longitudinal studies assessing RD changes post-treatment and correlating RWT with constipation duration could further enhance ultrasound’s diagnostic and monitoring utility.

## Conclusions

Our study demonstrates that transabdominal ultrasound measurement of transverse RD is a valuable non-invasive tool for diagnosing fecal impaction in children with FC, effectively distinguishing those with impaction from those without. Age-stratified analysis revealed that RD thresholds perform better in older children, offering high specificity and predictive accuracy, while showing moderate discrimination ability in younger children, highlighting the need for age-specific cut points. In contrast, rectal wall thickness did not contribute significantly to diagnosing fecal impaction, suggesting its limited clinical utility. These results position ultrasound as a promising, patient-friendly diagnostic adjunct in pediatric constipation management, with further refinement of age-specific thresholds needed to optimize its application across all age groups through large multicentric studies.
